# Biomarkers for Basal-like Breast Cancer

**DOI:** 10.3390/cancers2021040

**Published:** 2010-05-28

**Authors:** Jennifer R. Choo, Torsten O. Nielsen

**Affiliations:** Department of Pathology and Laboratory Medicine, University of British Columbia, Vancouver, Canada; E-Mail: jennchoo@interchange.ubc.ca

**Keywords:** breast cancer, basal-like, biomarkers, intrinsic subtype, immunohistochemistry, triple-negative, basaloid, expression profile

## Abstract

Initially recognized through microarray-based gene expression profiling, basal-like breast cancer, for which we lack effective targeted therapies, is an aggressive form of carcinoma with a predilection for younger women. With some success, immunohistochemical studies have attempted to reproduce the expression profile classification of breast cancer through identification of subtype-specific biomarkers. This review aims to present an in depth summary and analysis of the current status of basal-like breast cancer biomarker research. While a number of biomarkers show promise for future clinical application, the next logical step is a comprehensive investigation of all biomarkers against a gene expression profile gold standard for breast cancer subtype assignment.

## 1. Introduction

Gene expression profiling has enabled the classification of breast cancers into intrinsic molecular subtypes with diverse clinicopathologic features [1,2,3]. Representing ~15% of these biologically and clinically distinct forms of breast cancer [4,5,6], basal-like carcinomas are of particular interest to clinicians and researchers due to their characteristically poor prognosis and resistance to existing molecularly-targeted treatment modalities, such as endocrine therapy (e.g., tamoxifen and aromatase inhibitors) for hormone receptor-positive disease or trastuzumab for human epidermal growth factor receptor-2 (HER2)-positive disease, leaving cytotoxic chemotherapy as the principal systemic treatment [7,8,9,10]. Furthermore, younger women have a greater tendency to be afflicted by this aggressive subtype [11,12,13]. These clinically defining attributes of most basal-like carcinomas illustrate that, while remarkable strides are being made in our understanding of both molecular and genetic mechanisms of breast cancer, characterization of the basal-like subtype is a critical focus for clinical translation as both new diagnostics and treatments are needed. 

Basal-like breast carcinomas exhibit a certain degree of heterogeneity in their expression profile, morphology, immunophenotype, prognosis and treatment response. Basal-like gene expression profiles and biomarker expression are seen not only in typical high grade infiltrating ductal carcinomas, but also in metaplastic [14], secretory [15], medullary [16] and prognostically favorable adenoid cystic carcinomas [17]. Nevertheless, Weigelt *et al.* [18] recently provided strong support for the concept of basal-like breast cancer by illustrating that it is the intrinsic subtype most consistently identifiable by gene expression profile analysis. Multiple studies using different platforms and methodologies have revealed that basal-like breast carcinomas are generally characterized by negative expression of hormone receptors and related genes, as well as positive expression of a set of genes typically associated with basal epithelial cells, such as basal cytokeratins, P-cadherin, β4 integrin and nestin, in addition to several genes involved in cellular proliferation [1,2,19]. Given such consistencies in gene expression patterns, Stingl and Caldas [20] proposed that molecular characterization of breast cancers may provide insight into their cellular origins. However, the histogenesis of basal-like breast carcinomas is a controversial topic with some researchers believing these cancers develop from a multi-potential early precursor cell and others supporting the theory that such breast carcinomas arise from partially-committed luminal progenitor cells [21,22,23]. Simple studies on the preferential expression of basal versus luminal cytokeratins have been unable to provide sufficient support for either hypothesis [24], leading to extensive research efforts currently underway to find an answer. 

As histology is not consistently distinctive, and large scale expression profiling is not readily applied in a clinical setting, the search for clinically-practical cancer biomarkers that act as surrogate measurements of disease states is rapidly expanding in support of the paradigm shift towards more personalized medicine in this post-genomic era [25]. From improving the accuracy of diagnosis or risk assessment (via prognostic biomarkers) to guiding selection of optimal therapeutic interventions (via predictive biomarkers) [8,26,27,28], the potential clinical applications of these biological indicators are widely recognized in breast cancer research as having the potential to revolutionize patient management. Biomarkers for basal-like breast cancer are a subject of active and intense investigation for accurate diagnosis and therapeutic targets. The aim of this review is to evaluate our current state of knowledge on basal-like biomarkers as well as provide a critical survey of candidate biomarkers of basal-like breast cancer drawn from literature, followed by recommendations for future research directions. Particular emphasis is placed on immunohistochemical biomarkers confirmed on clinical biopsy and excision samples, as opposed to breast cancer cell lines, explanted or animal models.

## 2. Biomarker Panels for Basal-like Breast Cancer

Tumor biomarkers can encompass a range of molecules from nucleic acids to metabolites. However, protein biomarkers convert most readily into targeted therapies (as most pharmaceuticals tend to target proteins) and clinical diagnostic assays using standard existing platforms [28,29]. For instance, practical and clinically-accessible immunohistochemical techniques used routinely for breast cancer patient management are predominantly applicable to protein biomarkers. Despite semi-quantitative results and susceptibility to both inter-observer and technical variation—short comings which can be remedied to some extent through sufficient training and implementation of stringent protocols—immunohistochemistry is suited for application on formalin-fixed paraffin-embedded samples and also provides the unique advantage of simultaneous morphological analysis. In fact, few published reports on basal-like breast cancer have used a gene expression profile gold standard, opting instead to use immunohistochemistry-based methodologies while investigating this subtype primarily due to cost, but also to avoid the complex sample preparation and data analysis processes [5,30]. 

Further establishing immunohistochemistry as a cornerstone in breast cancer research, tissue microarrays enable highly efficient processing of hundreds to thousands of archival tumor specimens at one time with even more significant cost savings [31,32]. Facilitated by this relatively new technology, biomarker development has excelled to the point where prospectively-designed re-analyses of existing material (such as samples from completed clinical trials with extensive follow-up), sometimes termed ‘retrospective-prospective studies’, are beginning to generate clinically meaningful data about predictive biomarkers, and have the potential to change medical practice more quickly than new clinical trials [31,33,34,35]. Even so, finding a single biomarker that fulfills all requirements necessary to be deemed applicable in routine analysis has proven to be extremely difficult for basal-like carcinomas. More realistically, panels of breast cancer biomarkers can be designed in anticipation of phenotypic heterogeneity, and some have emerged with sufficient sensitivity and specificity for use in prognostication or prediction of treatment response in the clinical setting.

### 2.1. Current Examples: Immunohistochemical Definitions of Basal-like Breast Cancer

Immunohistochemical analysis of HER2 as well as estrogen and progesterone receptors (ER and PR, respectively) is used for predictive purposes in routine breast cancer patient management. Lack of expression of all three of these biomarkers predicts non-response to available endocrine (tamoxifen, aromatase inhibitors) and anti-HER2 (trastuzumab) targeted therapies, and has become known as a triple-negative phenotype (TNP). With approximately 70–90% of triple-negatives revealed to be basal-like breast carcinomas [36,37], TNP has been frequently used as a surrogate for the basal-like subtype. However, despite considerable overlap in behavioral/biological characteristics, several studies have shown that triple-negative and basal-like breast tumors are not synonymous, differing in prognosis and possibly in chemotherapeutic sensitivity [12,38,39,40,41]. Furthermore, an all-negative definition has a high propensity to mis-assign tumor classifications when biomarkers are negative for technical reasons. This and similar lines of evidence have prompted most researchers to acknowledge a distinction between TNP and basal-like tumors, leading to scrutiny of the validity of the triple-negative definition for basal-like breast cancer [12,38,42,43]. The primary reason for the continued use of a TNP category of breast cancers is its simplicity and convenience (based solely on information that can be readily extracted from a patient’s chart), and its identification of a specific group of breast cancers for which current targeted therapies are not expected to provide benefit. 

**Figure 1:**
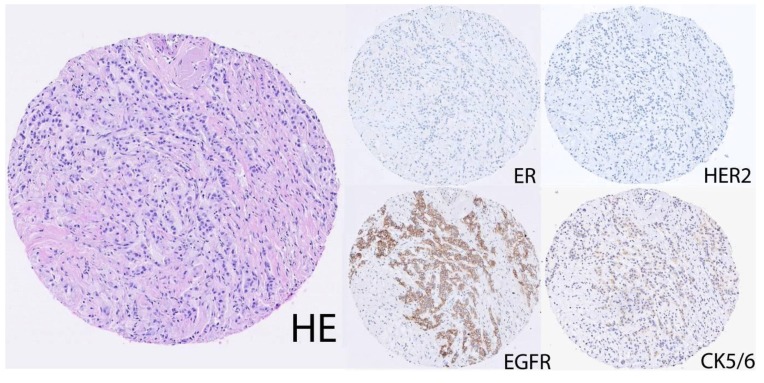
Immunohistochemical staining of a representative basal-like breast cancer using the definition of Nielsen *et al.* [5]. The case illustrated is ER negative, HER2 negative, EGFR strong positive and CK5/6 weak positive.

Another approach used to identify basal-like breast carcinomas is based on positive expression of basal cytokeratins (CKs) 5, 14 and 17 [6,13,44,45,46]. Prior to the discovery of the breast cancer intrinsic molecular subtypes, a number of studies had confirmed an association between poor prognosis and basal cytokeratin expression [1,47,48,49,50,51]. Subsequent research undertaken in light of the molecular identification of the basal-like subtype has greatly extended our knowledge of the clinical features associated with expression of basal cytokeratins in breast cancer [6,13,46], and these biomarkers have been used alone or incorporated into several immunohistochemical panels for identifying basal-like breast carcinomas. For instance, by comparing gene expression profiles to immunohistochemical results obtained using technically well-established antibodies, Nielsen *et al.* [5] defined basal-like breast cancer as any staining with CK5/6 or epidermal growth factor receptor (EGFR) antibodies in the context of HER2 and ER negativity (Figure 1). With a reported 76% sensitivity and 100% specificity for the basal-like subtype this definition has been widely used, and it has since been modified to incorporate PR-negativity to form a 5-marker immunopanel with greater prognostic value than the TNP definition for basal-like breast cancer [12]. Similarly, Livasy *et al.* [52] reported that negative expression of ER and HER2 with positive expression of EGFR, CK5/6, CK8/18 and vimentin was the most consistent immunophenotype of basal-like breast tumors. Also, yet another surrogate immunopanel with 78% sensitivity and 100% specificity against a gene expression profile gold standard was recently reported by Thike and colleagues [53], consisting of CK14, EGFR and a mixture of high molecular weight basal cytokeratins (using the pan-basal cytokeratin monoclonal antibody 34βE12).

### 2.2. Lack of a Consensus

The seminal paper by Perou and colleagues [1] that first elucidated the molecular portrait of breast cancer is largely responsible for inspiring interest in the basal-like subtype, catalyzing biomarker research for the purpose of recapitulating the gene expression classification as well as characterizing the biological and clinical features of this form of breast cancer. Although expression profiling can be considered a gold standard for identification of basal-like breast cancer, whether performed using microarrays or newer sequencing-based approaches such as the PAM50 qRT-PCR assay [54], it is currently not a feasible approach for large-scale application on routine formalin-fixed paraffin-embedded clinical samples (required for both retrospective application on large sample cohorts with long term follow-up and prospective application in standard hospital pathology laboratories). For this reason, immunohistochemical surrogates have become an important alternative. Major definitions are negative (*i.e.,* ER/HER2 double- or triple-negative incorporating PR), positive (e.g., purely based on basal cytokeratin expression), or a combination. Unfortunately, variable immunohistochemical definitions have impeded consistency in the interpretation of retrospective studies and confounded proposals for prospective implementation. In general, incorporating additional biomarkers into a panel can increase specificity, at the potential cost of sensitivity. Many biomarkers have been associated with the basal-like phenotype, and those with high sensitivity and/or specificity could improve the performance of immunohistochemical surrogate panels.

## 3. Biomarkers Associated with a Basal-like Breast Cancer Phenotype

In effort to develop clinical tests that more reliably diagnose this aggressive subtype of breast cancer and/or best define an entity that may have predictive value for treatment selection, many studies have been published over the last decade describing additional biomarkers that correlate with a clinical triple-negative phenotype, established immunohistochemical surrogates for basal-like breast cancer, or in some cases a microarray gold standard definition. Ranging from structural proteins to those involved in cellular processes, such as signal transduction and apoptosis, the proposed biomarkers for the basal-like subtype are diverse in class and function.

### 3.1. Structural

Structural proteins reported to have increased expression in basal-like breast carcinomas include basal cytokeratins (CK5/6, CK14 and CK17) [1,13,44,46,47,55], vimentin [52,56,57,58], fascin [58,59,60], nestin [61,62,63] and moesin [64,65]. Such cytoskeletal components are favorable antigens for immunohistochemical detection because of their stable and abundant cellular expression. Specificity can be an issue as these proteins are expressed in many cell types including benign breast epithelial and stromal elements, and so generally must be interpreted in their morphological context by an experienced pathologist. Most are still under investigation and have yet to provide actual clinical utility beyond that initially reported in the original publications, with the exception of basal cytokeratins which are perhaps the oldest and most clinically characterized biomarkers of basal-like breast carcinomas. For instance, Kusinska *et al.* [66] recently sought to address whether the inclusion of vimentin in an immunopanel consisting of ER, PR, HER2, and basal cytokeratins (CKs 5/6, 14 and 17) would better delineate their basal-like breast cancer definition (*i.e.,* TNP combined with basal cytokeratin positivity) using overall survival as the primary endpoint. In their analysis, it was determined that vimentin did not contribute prognostically to the immunopanel, which somewhat contradicts the results of Livasy *et al.* [52] mentioned previously while discussing current immunohistochemical definitions of basal-like breast cancer [66]. 

Playing a different type of structural role within the cell, claudins are protein components of tight junctions responsible for maintaining cell polarity and establishing a paracellular barrier that controls the ionic permeability of epithelial tissues [67]. Preferential expression of claudins 1 and 4 has been associated with poor prognosis and basal-like breast cancer [68,69,70]. Of a related nature (as structural components of plasma membrane caveolae), increased expression of both caveolin 1 and 2 has also been linked to the basal-like subtype [71,72,73,74]. A clear resolution of the role of the caveolins as basal-like biomarkers is hindered by controversy regarding their cellular distribution in invasive breast cancer, as well as recent evidence indicating a potentially stronger prognostic role for stromal rather than tumor cell expression of caveolin 1 [75,76].

### 3.2. Extracellular Interactions & Signal Transduction

Cell-cell and cell-extracellular matrix contacts mediate signaling cascades that culminate in diverse molecular responses pivotal to cancer, including angiogenesis, cell division, apoptosis, invasion and metastasis [77]. Some reported basal-like biomarkers are proteins normally secreted into the extracellular matrix, such as osteonectin (also known as SPARC) and osteopontin. These bone matrix-associated factors do not have well-defined primary roles in breast structure [58,78,79,80]. Osteopontin is a phosphorylated glycoprotein found in all body fluids, but its overexpression in the tumor cells of breast and other cancers has led to its investigation as a potential biomarker and anti-metastatic therapeutic target [81,82]. A study by Wang *et al.* [37] in which mean osteopontin levels were found to be significantly higher in triple-negative relative to non-triple-negative breast carcinomas provides preliminary evidence of a possible association with basal-like breast cancer. 

The laminin family of extracellular matrix glycoproteins involved in cellular adhesion has been associated with the basal-like subtype [57]. Specifically, high expression of laminin 5 (more recently referred to as laminin 332) has been observed in a variety of tumors including basal-like breast carcinomas [83,84]. A cell surface interacting partner for most laminins is α6β4 integrin, which is known to modulate signaling pathways involved in proliferation and survival [85,86]. Interestingly, Lu *et al.* [86] reported that the β4 integrin subunit is preferentially expressed in basal-like breast cancer compared to non-basal-like. Other cell surface molecules that are reported to exhibit increased expression in the basal-like subtype include nerve growth factor receptor (NGFR) [87], CD109 [88], placental cadherin (P-cadherin; P-CD) [4,89,90,91], CD44 [92,93], CD280 (also known as Endo180) [43,94], c-Met [64,95,96] and CD146 (also known as melanoma cell adhesion molecule) [95,97]. Illustrating the necessity for standardization of the methods and approaches used for biomarker analysis, contradictory evidence suggesting a breast tumor inhibitory role for CD146 has also been reported [98,99]. As noted by Ouhtit and co-workers [98], one of the original immunohistochemical studies that correlated CD146 overexpression with the basal-like subtype of breast cancer considered normal CD146 endothelial cell staining alone within tumor tissue as a CD146-positive tumor, which undoubtedly influenced the findings of the paper. In spite of this, a recent study by Zabouo *et al.* [97] confirmed a statistically significant association between CD146 and basal-like versus non-basal-like breast cancer as defined by a gene expression profile gold standard.

A select number of players involved in signal transduction have shown enough promise in both breast and other types of cancer to warrant major efforts in rational drug design. EGFR [5,100,101,102,103], c-Kit (also known as CD117) [5,17,102] and vascular endothelial growth factor (VEGF) [104,105] are candidate biomarkers of basal-like breast cancer with targeted therapies in development. Examples of possible biology-based therapeutic options available for treatment of basal-like breast cancer include anti-EGFR monoclonal antibodies (cetuximab and panitumumab), EGFR tyrosine kinase inhibitors (gefitinib, erlotinib and lapatinib), imatinib for c-Kit kinase inhibition and bevacizumab for VEGF inhibition [106,107,108,109,110]. Initial trials in breast cancer have been somewhat disappointing with only moderate clinical efficacy; the described biomarkers may not be sufficiently robust to provide predictive value [111,112,113,114,115]. However, the relatively prevalent overexpression of EGFR, c-Kit and VEGF among basal-like and triple-negative breast cancers has maintained hope that current trials underway restricted to these tumor types will maximize the chance of identifying a responsive subgroup of breast cancer and offer more definitive data [104,105,116,117].

### 3.3. Transcription, Cell Cycle Regulation and DNA Damage Repair

While the cell surface components that govern tumor interactions have gained extra attention because they are targeted by several new pharmaceuticals, common downstream signaling effects culminating in transcriptional activation and subsequent dysregulation of a multitude of cellular processes, represent more consistent driving forces of oncogenesis [78,118]. As might be expected, several transcription factors have been demonstrated to be preferentially expressed in basal-like breast cancer including c-Myc [43], Sox2 [56,119], FOXC1 [120,121], FOXC2 [122], E2F-5 [123], YB-1 [103,124,125], p-JNK [126], p63 [2,4,127] and p53 [2,11,128,128]. It is important to note that both overexpression and mutation of p53 are collectively seen in more than 85% of basal-like breast tumors [130,131,132]. Given that p53, as the “guardian of the genome”, is a critical tumor suppressor protein, its mutation or deletion is commonly observed in many aggressive cancer types [133,134]. With regards to refining the immunohistochemical classification of basal-like breast cancer, the clinical utility of p53 in routine analysis may be limited since the specific location and type of mutation in the protein was recently shown to influence clinical outcome in breast cancer patients [135]. Consequently, while p53 accumulation is considered a classic indicator of its mutation status, the best approach for p53 analysis is likely a more technically-demanding combination of immunohistochemistry and genetic screening techniques as recently illustrated by Manie *et al.* [129].

Being key players involved in regulation of the cell cycle, biologically plausible reports of p16, Skp2 and cyclin E overexpression in basal-like breast cancer have been made [45,130,136,137,138,139,140,141]. Interestingly, a tissue microarray study by Voduc and colleagues [138] that investigated the combinatorial overexpression of Skp2 and cyclin E in breast cancer was able to confirm the association with the basal-like subtype and prognostic significance in univariable but not multivariable analysis (taking into account standard clinicopathologic variables, such as patient age, tumor size, tumor grade and nodal status). Such results, possibly attributable to a lack of statistical power, may simply suggest that the combination of Skp2 and cyclin E would be more useful as part of a larger immunopanel [138]. Similarly, Ki67 (involved in rRNA synthesis and other unidentified cellular functions) is an established marker of proliferation associated with the basal-like subtype and poor prognosis [59,142,143]. However, the high Ki67 index characteristic of high grade and ER negative tumors renders it prognostically insignificant within the basal-like subtype of breast cancer [144,145].

Although of controversial value as an immunohistochemical biomarker, the relationship between *BRCA1* status and basal-like breast cancer could potentially lead to great progress in the field. Deficiency of normal BRCA1-mediated double-stranded DNA repair function predisposes female carriers to early development of ovarian cancer and, more commonly, to a highly aggressive form of breast carcinoma [146]. Relating back to the earlier discussion of p53, a strong correlation between *BRCA1*-related hereditary breast cancer and the basal-like subtype was initially made based on preferential overexpression of p53 and other phenotypic similarities [19,147]. Furthermore, studies have demonstrated disruption of BRCA1 through epigenetic and other regulatory mechanisms, which may occur in sporadic cases of basal-like or triple-negative breast cancer [148,149,150]. Several immunohistochemical and gene expression profiling studies have shown that BRCA1-deficient tumors possess many biological and molecular characteristics of basal-like breast cancer [3,19,79,130,151,152]. Correspondingly, the manifestation of a basal-like phenotype by BRCA1 inactivation has been shown in breast cancer cells [153,154] and corroborated in animal studies by multiple groups, providing further support for the association between BRCA1 pathway disruption and basal-like breast carcinomas [155,156]. Exploitation of this relationship, provides the rationale to target basal-like breast tumors using inhibitors of poly [ADP-ribose] polymerase 1 (PARP1; involved in single-stranded DNA break repair), since it is postulated that pharmaceutical inhibition of PARP1 in combination with the pre-existing DNA repair dysfunction from BRCA1 deficiency underlies a situation of synthetic lethality for tumor cells, yet minimal toxicity to normal cells [146]. Clinical trials of PARP1 inhibitors in *BRCA1*-related and/or triple-negative breast cancers are currently underway to address this hypothesis [157,158,159], and preliminary results in ovarian cancer have been promising [160,161].

### 3.4. Biomarkers of Miscellaneous Function

A summary of reported biomarkers of basal-like breast cancer can be found in Table 1, which also includes several proposed biomarkers that did not clearly fall into the above categories. While less well-defined, increased expression of insulin-like growth factor mRNA binding protein 3 (IMP3) [162], aldehyde dehydrogenase 1 (ALDH1) [163,164], aquaporin 1 (AQP1) [165], basal/myoepithelial markers belonging to the S100 family of proteins (A2, A8 and A9) [80,83,86,166,167], organic anion transporting polypeptide 2 (OATP2) [10], phosphohistone H3 [168] and the multidrug-resistance pump P-glycoprotein [169] have each been associated with basal-like and/or triple-negative breast cancers. In addition, the recently observed overexpression of carbonic anhydrase IX (CAIX; CA9) in basal-like carcinomas supports what has long been suspected, that tumors of this subtype activate a hypoxic response to survive under conditions of rapid and aggressive growth [40,170]. As noted by Lancashire *et al.* [170], the focal expression of CAIX (and any marker with a similar expression pattern) requires immunohistochemical assessment on whole tissue sections as opposed to tissue microarray cores for optimal detection. Finally, fatty acid binding protein 7 (FABP7) [10,43,171] and αB-crystallin [172,173] have preferentially higher expression in the basal-like subtype at both the protein and DNA level making them prime candidates for further investigation as biomarkers of basal-like breast cancer [1,2,19]. 

**Table 1:** Candidate immunohistochemical biomarkers of basal-like breast cancer.

Biomarker	Experiment Format	Basal-like Definition	Frequency Among Basal-like (%)	Frequency Among Non-basal-like (%)	Literature References
**Vimentin**	TMA	Combined	21/27 (78)	30/194 (16)	[57]
**Fascin**	TMA	Combined	14/26 (54)	43/198 (22)	[59]
**Nestin**	Whole sections	TNP	12/21 (57)	12/129 (9)	[61]
	Whole sections	TNP	14/16 (88)	0/32 (0)	[62]
	TMA	Combined	15/22 (68)	3/143 (2)	[63]
Moesin*	TMA	Combined	23/28 (82)	14/64 (22)	[65]
**Claudin 1**	TMA	Combined	11/18 (61)	ND	[68]
**Claudin 4**	TMA	Combined	34/38 (90)	42/66 (64)	[69]
**Caveolin 1**	TMA	Combined	17/53 (32)	25/314 (8)	[71]
	TMA	Combined	11/53 (21)	10/435 (2)	[72]
	Whole sections	Combined	21/30 (70)	1/202 (0)	[73]
Caveolin 2*	TMA	Combined	10/50 (20)	5/270 (2)	[71]
	TMA	Combined	11/28 (39)	1/173 (0)	[74]
Osteopontin*	Whole sections	TNP	ND	ND	[37]
**Laminin**	TMA	Combined	11/26 (42)	28/193 (15)	[57]
**β4 Integrin**	Whole	TNP	15/27 (56)	18/71 (25)	[86]
NGFR**	TMA	Combined	10/33 (30)	1/190 (0)	[87]
**CD109**	Whole sections	TNP	18/30 (60)	0/53 (0)	[88]
**P-cadherin**	TMA	Combined	10/12 (83)	34/128 (27)	[4]
	Whole sections	Combined	6/8 (75)	13/68 (19)	[90]
**CD44 (high)**	TMA	Combined	20/23 (87)	61/141 (43)	[93]
**OATP2**	TMA	Basal CK	23/161 (14)	20/394 (5)	[10]
CD280*	TMA	Combined	6/28 (21)	2/175 (3)	[94]
	TMA	Combined	9/66 (14)	11/302 (4)	[94]
**CD146**	TMA	TNP	25/76 (33)	13/425 (0)	[97]
EGFR*	TMA	GEP	41/93 (44)	41/521 (8)	[5]
	Whole sections	TNP	163/284 (57)	ND	[101]
**c-Kit**	TMA	Basal CK	32/102 (31)	67/605 (11)	[5]
**VEGF**	Whole sections	Basal CK	15/54 (28)	4/46 (9)	[105]
**Sox2**	TMA	Combined	13/30 (43)	16/147 (11)	[119]
**FOXC1**	TMA	Combined	ND	ND	[120,121]
**FOXC2**	TMA	NS	8/18 (44)	4/99 (4)	[122]
**E2F-5**	Whole sections	TNP	14/27 (52)	5/30 (17)	[123]
	Whole sections	Combined	14/25 (56)	5/32 (16)	[123]
**YB-1**	TMA	TNP	27/37 (73)	ND	[103]
**p-JNK**	Whole sections	Combined	16/25 (64)	59/134 (44)	[126]
**p63**	TMA	Combined	6/11 (56)	24/137 (18)	[4]
	Whole sections	Basal CK	13/19 (68)	3/83 (4)	[127]
**p53**	Whole sections	Basal CK	7/19 (37)	28/83 (34)	[127]
	TMA	TNP	13/32 (41)	44/103 (43)	[128]
	Whole sections	Basal CK	32/95 (34)	27/151 (18)	[130]
	Whole sections	Basal CK	25/49 (51)	100/278 (36)	[169]
**p16 (strong)**	Whole sections	GEP	22/33 (69)	10/86 (12)	[140]
Cyclin E*	Whole sections	Basal CK	41/92 (45)	22/150 (15)	[130]
**Ki67**	TMA	Combined	6/11 (55)	27/125 (22)	[4]
	Whole sections	Basal CK	15/19 (79)	32/83 (39)	[127]
	Whole sections	Basal CK	39/49 (80)	81/278 (29)	[169]
IMP3*	Whole sections	TNP	25/32 (78)	20/106 (19)	[162]
**ALDH1**	Combined	Combined	9/23 (39)	24/160 (15)	[164]
AQP1*	TMA	TNP	10/45 (22)	1/157 (0)	[165]
PPH3*	Whole sections	Combined	19/21 (90)	65/219 (30)	[168]
**P-glycoprotein**	Whole sections	Basal CK	29/49 (59)	85/278 (31)	[169]
CAIX*	Whole sections	Combined	16/62 (26)	43/394 (11)	[40]
FABP7**	TMA	Basal CK	43/155 (28)	40/393 (10)	[10]
	Whole sections	Combined	10/11 (91)	14/77 (18)	[171]
αB-crystallin*,**	TMA	Combined	18/40 (45)	17/288 (6)	[172]
	Whole sections	Combined	26/32 (81)	0/21 (0)	[173]

* Biomarker shown to be an independent prognostic factor. ** Distinguishes between good and poor prognostic groups within the set of basal-like tumors. TMA = Tissue microarray; Basal CK = defined by basal cytokeratin(s) positivity; TNP = Triple negative phenotype; Combined = defined as TNP plus a positive basal-like biomarker including, but not limited to, basal CKs; GEP = Gene expression profile; ND = No data; NS = Not specified

## 4. What Is Next in Basal-like Breast Cancer Biomarker Research?

For many biomarkers of basal-like breast cancer, validation studies (ideally from independent groups) are required, as sensitivity and specificity issues arising due to the use of multiple different cut-offs are unavoidable without the establishment of technical standards. Even the commonly-used triple negative definition suffers from these inconsistencies, as both 1% and 10% cut-offs for ER and PR are widespread. Consequently, the detection of basal-like breast cancers using the TNP definition yields variable results with a higher cut-off (e.g., 10%) increasing the sensitivity of the definition at the expense of specificity. Basal cytokeratins, nestin, caveolins 1 and 2, P-cadherin, EGFR and αB- crystallin are some of the better characterized basal-like biomarkers, yet most must still be considered to remain in a developmental phase, in which the data-driven cutpoint optimization used in a majority of the original studies requires validation. In turn, considerably more research is necessary before any single biomarker is ready for clinical application. 

Even where technical issues are worked out and clinical application is practical, clinical utility typically requires predictive, rather than merely prognostic value. Further adding to the lengthy introductory process of biomarkers into routine analysis, prospective randomized clinical trials are the usual route that must be taken to achieve practice-changing, Level 1 evidence, and such trials are few and very rarely conducted for predictive biomarkers. Existing breast cancer trials with completed long term follow-up were mostly designed prior to the recognition of the basal-like subtype. Nevertheless, using archived tissue specimens from completed clinical trials, the criteria to obtain Level 1 evidence to facilitate implementation of a biomarker into clinical practice was recently published by Simon *et al.* [34], in which a rigorous definition of assay methodology and interpretation, including an entirely pre-specified plan for statistical analysis, is mandatory. Initial work in this regard has suggested, for example, an association of the basal-like phenotype with benefits from adjuvant taxanes but not anthracyclines [35]. In addition, Cheang *et al.* [41] recently presented data suggesting that basal-like breast cancers derive superior benefit from cyclophosphomide-methotrexate-fluorouracil (CMF) over a more widely-employed anthracycline-based treatment regimen (cyclophosphomide-epirubicin-fluorouracil; CEF). In both these studies, use of a broader triple negative definition resulted in a loss of statistical significance, emphasizing how use of better biomarker panels and more precise definitions may be required to identify the best treatment for women with basal-like breast cancers. For example, an equal or superior response of basal-like breast carcinomas to non-anthracycline chemotherapies has implications for the delivery of optimal care for patients, since anthracycline-containing regimens (a standard therapy) have the potential for specific side effects, such as cardiotoxicity [174].

Understandably, candidate biomarkers selected to undergo the rigorous testing and validation process for approval must be chosen wisely for the sake of time, cost and preservation of valuable resources. To meet these challenges, a logical next step in basal-like breast cancer biomarker research is to determine the immunohistochemical sensitivity and specificity of as many proposed biomarkers as possible relative to a gene expression profile gold standard. A multi-marker immunohistochemical panel consisting of the best biomarkers could then be assembled to maximize sensitivity and specificity for basal-like breast cancer. After further evaluation on well-characterized breast tumor specimens from independent cohorts and clinical trials, the final product would be a thoroughly validated, practical and clinically-accessible assay suitable for novel applications in the management of basal-like breast cancer patients. 

While the described process may seem straightforward in concept, its successful execution is dependent on several factors requiring special consideration. First, standard and commercially-available immunohistochemistry-grade antibodies with demonstrated robustness and sensitivity should be used to ensure assay consistency. Moreover, staining protocols must be standardized and subject to quality control. A stringent approach is of great importance for an immunohistochemistry-based clinical assay since interpretation of results can be easily confounded seemingly due to minor procedural inconsistencies, such as using different antibody clones. In addition, monoclonal antibodies are preferred to polyclonals for their superior specificity and subsequently reduced background staining levels which, in turn, lead to decreased inter-observer and technical variation. This approach has been successful in lymphoma subtyping, for example [175]. Even so, it is possible that immunohistochemical approaches may not be up to the task, and gene expression measurements applicable to formalin-fixed paraffin-embedded specimens may provide the necessary means [54,176,177,178].

Second, access to a large collection of breast tumor specimens with extensive follow-up is a requirement for meaningful exploratory analyses, let alone for confirmation of the prognostic and predictive value of a biomarker panel. Unfortunately, despite abundance of formalin-fixed paraffin-embedded breast cancer samples in hospital archives, comprehensive outcome data is restricted to a limited number of large research institutions and cancer cooperative groups. Given the considerable sample size needed for sufficient statistical power, a tissue microarray platform is a useful technique to efficiently pursue such investigations. Once again, access to necessary resource tissues as well as the equipment and expertise for such tissue microarray research is limited. A third commonly underestimated factor in immunohistochemical assay development is the meticulous statistical evaluation and laborious model building that must be uniformly conducted with utmost care. Determining the best immunopanel will undoubtedly involve striking a balance between optimizing sensitivity/specificity and practical limits on the number of biomarkers in a panel [179].

Finally, other than a prospective clinical trial specifically designed to address the optimized immunopanel, the highest level of evidence that provides grounds for proposing a change in medical practice must be obtained from two or more consistent retrospective-prospective studies that illustrate the clinical utility of assessing combinatorial expression of the selected biomarkers that comprise the optimized immunopanel [34]. However, the value and scarcity of clinical trial specimens inherently leads to many restrictions on their use, and applications for tissue access can take months and even years to be approved. Nonetheless, these steps must be taken in order to identify the true value of basal-like breast cancer biomarkers and translate this into improved patient care, ultimately giving those afflicted by these aggressive carcinomas the best chance of a cure.

## 5. Conclusions

Basal-like breast cancer accounts for a disproportionately high number of breast cancer-related deaths [180], and due to limited treatment options and lack of targeted therapies will continue to present a significant clinical challenge until more effective interventions are discovered. However, before we can translate existing knowledge into medical diagnosis and treatment, a clinically-practical assay that can reliably identify basal-like carcinomas is necessary. Current immunohistochemical definitions for basal-like breast cancer have limited sensitivity and/or specificity, hindering implementation into routine analysis. This illustrates the need to refine the immunohistochemical classification or develop alternative approaches. Validated biomarkers with high sensitivity and/or specificity against a gene expression profile gold standard can then be used to either build a novel immunohistochemical classifier or improve the performance of existing definitions. The assembly of a definitive immunopanel that accurately identifies the basal-like subtype will enable research to be undertaken in a more significant and meaningful context with regards to basal-like breast carcinoma biology, prognosis and prediction of therapeutic response. This would allow optimal selection from current management options and facilitate efficient development of new targeted therapies.
